# Characterization of Cellulose Fiber Derived from Hemp and Polyvinyl Alcohol-Based Composite Hydrogel as a Scaffold Material

**DOI:** 10.3390/polym15204098

**Published:** 2023-10-16

**Authors:** Praewa Promdontree, Pakpoom Kheolamai, Artjima Ounkaew, Ravin Narain, Sarute Ummartyotin

**Affiliations:** 1 Department of Materials and Textile Technology, Faculty of Science and Technology, Thammasat University, Pathumthani 12121, Thailand; pwpraewapwp@gmail.com; 2 Division of Cell Biology, Department of Preclinical Sciences, Faculty of Medicine, Thammasat University, Pathumthani 12120, Thailand; kpakpoom@tu.ac.th; 3 Department of Chemical and Materials Engineering, University of Alberta, Edmonton, AB T6G 2G6, Canada; 4 Center of Excellence on Petrochemical and Materials Technology, Chulalongkorn University, Bangkok 10330, Thailand

**Keywords:** hemp, cellulose fiber, cellulose nanocrystals, polyvinyl alcohol, hydrogel composite, cytotoxicity

## Abstract

Cellulose nanocrystals (CNCs) were successfully extracted and purified from hemp using an alkaline treatment and bleaching process and subsequently used in conjunction with polyvinyl alcohol to form a composite hydrogel. Cellulose nanocrystals (1–10% (*w*/*v*)) were integrated into polyvinyl alcohol, and sodium tetraborate (borax) was employed as a crosslinking agent. Due to the small number of cellulose nanocrystals, no significant peak change was observed in the FT-IR spectra compared to pristine polyvinyl alcohol. The porosity was created upon the removal of the water molecules, and the material was thermally stable up to 200 °C. With the presence of cellulose nanocrystals, the melting temperature was slightly shifted to a higher temperature, while the glass transition temperature remained practically unchanged. The swelling behavior was examined for 180 min in deionized water and PBS solution (pH 7.4) at 37 °C. The degree of swelling of the composite with cellulose nanocrystals was found to be higher than that of pristine PVA hydrogel. The cell viability (%) of the prepared hydrogel with different proportions of cellulose nanocrystals was higher than that of pristine PVA hydrogel. Based on the results, the prepared composite hydrogels from cellulose nanocrystals extracted from hemp and polyvinyl alcohol were revealed to be an excellent candidate for scaffold material for medical usage.

## 1. Introduction

With the exponential growth of the worldwide population, concerns over the shortage of food, environmental issues, and healthcare are considered as some of the most important aspects nowadays. Various technologies have been extensively developed in order to solve these issues. One of the most important concerns is typically related to healthcare management. This can be employed as an indicator to assess the quality of life. Up to the present time, various technologies for healthcare management have been developed to improve quality of life, such as functional food, medical screening, and surveillance, as well as protection and/or care. However, in order to reach the target of excellence in healthcare management, cost is also an important factor to consider. It should be noted that high-quality healthcare is costly due to the necessity of imported medicine and medical supplies from overseas.

Hydrogel is considered as one of the most effective materials for medical uses. Hydrogel is typically defined as a three-dimensional polymeric network containing chemically or physically crosslinked hydrophilic polymer chains. It can also retain a large amount of water. Due to the presence of hydrophilic groups, it can be employed to absorb wound exudates and allow oxygen transportation in order to accelerate the wound healing process. Moreover, it can be designed based on a biocompatible concept in order to prevent bacterial infection and maintain moisture content, as well as support the adhesion to tissue [1,2]. Currently, the use of hydrogel provides various advantages for medical research, including the encouragement of an appropriate microenvironment for cell growth and the recruitment of fibroblasts, as well as the proliferation process [3]. Recently, Liang et al. [4] also designed functional hydrogels for wound healing. The hydrogels were evaluated for their skin wound healing efficacy and parameters such as antimicrobial properties, adhesion, and hemostasis, as well as anti-inflammatory properties, were investigated. Li et al. [5] also found that hydrogels can be designed as a drug delivery system providing sustained drug release of various therapeutic agents, including small-molecule drugs and macromolecules, as well as cells.

To abide by the environmental preservation policy, the design of hydrogel should preferably be from natural sources. Hydrogel composite prepared from bio-based materials is therefore considered as a promising route. Besides their eco-friendly nature, their ease of degradation as well as their non-toxicity are interesting attributes.

Therefore, hemp-based materials are regarded as important components of wound dressings. Hemp is considered a botanical class of “*Cannabis sativa*”, and it has been used in various applications, including paper, textiles, biodegradable plastic, and animal feed, as well as for medicinal use. From a structural point of view, cellulose is the main component of hemp (55–72 wt%). It is a polysaccharide consisting of linear chains of several hundreds to many thousands of β (1 → 4) linked by D-glucose units [6]. It has many outstanding properties, including a very large surface area-to-volume ratio, good mechanical properties, and a very low coefficient of thermal expansion [7]. In order to employ cellulose derived from hemp with higher efficiency, cellulose-based composite materials were developed. It can be used as a filler in order to enhance the dimensional stability of a polymer matrix. Recently, in 2021, Rukmanikrishnan et al. [8] developed composite hydrogels from polyvinyl alcohol (PVA), polyethylene oxide (PEO), and sodium carboxymethyl cellulose (NaCMC). These composite hydrogels showed excellent structural and mechanical properties. Furthermore, the hydrogels displayed good biocompatibility and non-cytotoxicity. In 2022, Revati et al. [9] developed a bioactive, biocompatible, and porous polylactic acid (PLA) reinforced with cellulose nanofiber (CNF) scaffolds. Reinforcement with CNF improved the biodegradation of the scaffolds. Doustdar et al. [10] developed scaffolds from chitosan/cellulose nanocrystals with different crosslinking methods. FESEM images revealed that all the scaffold samples are three-dimensional networks in which the pores are connected. The structure of the scaffold was found to be suitable for cell encapsulation, proliferation, and growth. Doostan et al. [11] developed electrospun polyvinyl alcohol and chitosan (PVA/CS) nanofibrous scaffold-loaded flaxseed extract. The prepared scaffold exhibited excellent mechanical properties and biodegradation, as well as high water absorption and porosity. In 2023, Sriwong et al. [12] fabricated cellulose-based scaffolds derived from sugarcane bagasse (SCB) with hydroxyapatite (HA). SEM images confirmed the correct porosities of the scaffolds for cellular activities. The prepared scaffolds exhibited a low degradation rate. Furthermore, the MTS tetrazolium assay and alkaline phosphatase (ALP) activity indicated that cellulose combined with HA scaffolds supported bone cell proliferation and differentiation. To encourage the use of hydrogels prepared from bio-based materials, composite hydrogel based on cellulose derived from hemp is considered as an attractive strategy.

In this research, we present the development of cellulose nanocrystal-based composite hydrogels for wound dressing applications. Cellulose nanocrystals were successfully extracted and purified from hemp and hydrolyzed by sulfuric acid. CNC-polyvinyl alcohol composite hydrogels were prepared. The physicochemical properties and swelling behavior in DI water and PBS solution of the hydrogels were measured, and cytotoxicity studies were conducted.

## 2. Experimental Section

### 2.1. Materials

The raw hemp fiber (*Cannabis sativa*) was obtained from a local farm in Thailand. It was ground and stored before use. Cellulose nanocrystals were successfully extracted from the hemp fiber. Cellulose nanocrystals were employed as a reinforcement material in polyvinyl alcohol-based hydrogels. PVA, M_w_ = 100,000 g/mol, was purchased from Chem-Supply, Pty, Ltd. (Gillman, Australia). Sodium hydroxide (NaOH) was purchased from Merck, Co., Ltd. (Bangkok, Thailand). Glacial acetic acid (CH_3_COOH) was purchased from M.S. Chemical Co., Ltd. (Bangkok, Thailand). Sodium chlorite (NaClO_2_) was purchased from DC Fine Chemicals, Ltd. (Enfield, UK). They were employed as chemical treatment reagents. Sulfuric acid (H_2_SO_4_) was purchased from QRëC™ (Quality Reagent Chemical), Co., Ltd. (Chonburi, Thailand) and was used as a chemical reagent to modify the cellulose structure. Sodium tetraborate (Na_2_B_4_O_5_(OH)_4_.8H_2_O) was purchased from Elago Enterprises, Pty, Ltd. (Cherrybrook, Australia). It was employed as a crosslinking agent. Phosphate-buffered saline (PBS; pH 7.4) of biotechnology grade was purchased from Amresco (Cleveland, OH, USA), Inc. All chemical reagents were used as received without further purification.

### 2.2. Methodology

#### 2.2.1. Extraction and Purification of Cellulose Fiber

The cellulose microfiber (CMF) was extracted from the hemp fiber with alkali treatment. The objective of this process is to remove non-cellulosic components such as hemicellulose and lignin. The experiment was based on the protocol of Zhao et al. [13] with modifications. Briefly, the raw hemp fiber was first dried at 65 °C for 24 h and ground using a nano-grinder (High Energy Ball Mills, Emax, Germany) to decrease its size. Subsequently, it was treated with 18% (*w*/*v*) NaOH solution at 80 °C for 2 h under mechanical stirring to remove impurities. The alkali-treated hemp fiber was bleached 2 times with acetate buffer. The buffer was prepared by adding 2.7 g of NaOH and 7.5 mL of glacial acetic acid into 100 mL of distilled water. Then, 2% (*w*/*v*) NaClO_2_ was poured into the mixture, and the solution was stirred at 80 °C for 2 h. Next, the bleached hemp fiber was washed with distilled water until a neutral pH was achieved, and the samples were stored in the form of a suspension. Additional information is shown in our previous report [14].

#### 2.2.2. Extraction of Cellulose Nanocrystals (CNCs) with Sulfuric Acid Hydrolysis

Cellulose nanocrystals (CNCs) were successfully extracted with sulfuric acid hydrolysis based on the guidance of Leong et al.’s [15] protocol. The main objective of acid hydrolysis is to break the glycosidic bonding. Briefly, the CMF was treated with 0.9 M sulfuric acid solution at 50 °C for 15 min under mechanical stirring (ratio: 1 g/20 mL) (IKA Eurostar 20 Digital Overhead Stirrer, Bangkok, Thailand). Then, the obtained CNCs were quenched with cold distilled water for 15 min using a probe-type ultrasonic homogenizer (28% amplitude). After that, the produced CNCs were washed 3 times for 5 min with successive centrifugations until a neutral pH was reached. The centrifugation was set to a rotation speed of 900 rpm for 5 min. The cellulose nanocrystals were successfully extracted from the plant with high purity.

#### 2.2.3. Preparation of Cellulose Nanocrystal and Polyvinyl Alcohol-Based Composite Hydrogel

The cellulose nanocrystal and polyvinyl alcohol-based composite hydrogel was successfully prepared. Firstly, 5% (*w*/*v*) polyvinyl alcohol was dissolved in deionized water at 85 °C for 1 h under mechanical stirring. Subsequently, 1, 3, 5, 7, and 10% (*w*/*v*) of cellulose nanocrystals were added into a polyvinyl alcohol solution and continuously stirred to achieve a homogeneous solution. Then, 1% (*w*/*v*) of sodium tetraborate was dissolved in deionized water. The mixture was then cooled down to room temperature. The sodium tetraborate solution was added into the mixture and continuously stirred to form hydrogel. The hydrogel samples were stored in a refrigerator at 3 °C. To prepare freeze-dried forms, the hydrogel samples were stored in a freezer at −40 °C for 24 h. Subsequently, the samples were put in a freeze-dryer (CoolSafe Touch 100-9, ScanVac, Allerod, Denmark) at −76 °C in a vacuum of 0.001 mbar for 48 h. The freeze-dried samples were kept in a desiccator to prevent moisture absorption.

#### 2.2.4. Swelling Behavior

All composite hydrogels were investigated for their swelling behavior by using the gravimetric technique. Samples were cut into equal sizes and weighed (W_dry_). The samples were immersed in a deionized water and phosphate-buffered saline (PBS; pH 7.4) solution at 37 °C for 3 h. The weights of samples were measured every 30 min. Six samples were removed from the solution and dried with filter paper (W_wet_). The data were reported as the statistical average and standard deviation. The swelling ratio (%) was determined as follows:

$$\mathrm{Swelling}\;\mathrm{degree}\;\left(\%\right)=\left(\frac{W_{\mathrm{wet}}-W_{\mathrm{dry}}}{W_{\mathrm{dry}}}\right)\times 100$$

where W_wet_ is the weight of swollen hydrogel and W_dry_ is the initial weight of the freeze-dried hydrogel.

#### 2.2.5. Gel Fraction

All compositions of prepared freeze-dried cellulose nanocrystal polyvinyl alcohol-based composite hydrogel were cut into similar sizes and weighed (W_i_). The dried samples were immersed in deionized water. After a sufficient time, the specimens were dried to a constant weight in an oven at 60 °C for 24 h (W_d_). The specimens were measured, and the data were reported as the statistical average and standard deviation. The gel fraction percentage (GF%) was defined as follows:

$$\mathrm{GF}\%=\left(\frac{W_{d}}{W_{i}}\right)\times 100$$

where W_d_ is the weight of swollen hydrogel after drying in an oven to a constant weight and W_i_ is the initial weight of the freeze-dried hydrogel.

#### 2.2.6. Cytotoxic Test

The prepared freeze-dried hydrogel composite was studied via an extract test using human dermal fibroblasts, adult (HDFa) and cancer cells (Hela), as model cells. The samples were prepared in wells of a 24-well plate (Costar, Corning, Corning, NY, USA). For this purpose, the composite hydrogels in the 24-well plate were sterilized with UV exposure for 30 min before culturing. Then, the previously sterilized hydrogel samples were incubated with the culture medium composed of stock DMEM/F12, FBS (10%), and pen/strep (1%) (~200 mg per 1 mL of medium) at 37 °C, and supernatants (extracts) were collected after 24 h. The HDFa and Hela cells were placed into wells of a 96-well plate at a density of 1 × 10^4^ cells/well and cultured in complete medium and incubated in a CO_2_ incubator (37 °C, 5% CO_2_) for 24 h. The medium in each well was replaced with different concentrations and volumes at 25, 50, and 100 µL of the extracts, and the cells were proliferated under a 37 °C humidified atmosphere containing 5% CO_2_ for 24 h. The blank without composite hydrogel was set as a control. The cytotoxicity test was considered with a 3-(4,5-dimethylthiazol-2-yl)-2,5-diphenyltetrazolium bromide (MTT) cytotoxicity assay. After 2–4 h of incubation in MTT solution (0.5 mg/mL PBS), the active cells were stained with MTT. The MTT was removed; then, dimethyl sulfoxide (DMSO) was typically added to each well. Each well became purple due to the DMSO dissolving purple formazan crystals in viable cells. The cytotoxicity test was quantified using a microplate reader at an absorbance of 570 nm.

$$\mathrm{Cell}\;\mathrm{viability}\;(\%)=\left(\frac{{\mathrm{OD}}_{570\;\mathrm{sample}}}{{\mathrm{OD}}_{570\;\mathrm{blank}(\mathrm{control})\;}}\right)\text{×}\;100$$

### 2.3. Instruments

#### 2.3.1. Fourier-Transform Infrared Spectroscopy

The sample was prepared in gel form and subsequently analyzed using FT-IR spectroscopy (Nicolet Impact 410 FT-IR) at room temperature. The FT-IR spectra were recorded in the spectral range 4000–400 cm^−1^ with a resolution of ±4 cm^−1^ and a scan frequency of 32 times.

#### 2.3.2. X-ray Diffraction

The crystallinity of the composite was determined using X-ray diffraction (Bruker AXS Model D8 Advance, Mannheim, Germany). The sample was employed using nickel-filtered Cu Kα radiation at 40 kV and 40 mA. The patterns were recorded between 5 and 50°.

#### 2.3.3. Scanning Electron Microscopy

The composite was fractured in liquid nitrogen in order to observe the cross-section. The sample was coated with gold by a sputtering device (QUORUM Q150R ES, Hertfordshire, UK) at 23 mA for 45 s. Then, scanning electron microscopy (JEOL JSM7800F, Tokyo, Japan) was used to photograph the morphology of the hydrogel composite at 100× magnification and 2 kV of accelerating voltage.

#### 2.3.4. Thermogravimetric Analysis

The thermal stability of the composite was investigated with TGA (TA TGA55, New Castle, DE, USA). In total, 5 mg of sample was placed in an aluminum pan. It was heated from 30 °C to 600 °C in N_2_ with a heating rate and a flow rate of 10 °C/min and 60 mL/min, respectively.

#### 2.3.5. Differential Scanning Calorimetry

The thermal behavior of the composite was characterized using DSC (Mettler Toledo, DSC 3+, Greifensee, Switzerland). The sample was placed in an aluminum pan (sample holder). DSC of the composite hydrogel was employed from room temperature to 400 °C in N_2_ with a heating rate of 10 °C/min. The characteristic temperatures were determined from the heat flow curve as the glass transition temperature, melting temperature, and specific heat capacity.

## 3. Results and Discussion

### 3.1. Characterization of Cellulose Nanocrystals from Hemp-Based Fiber

**Figure 1:**
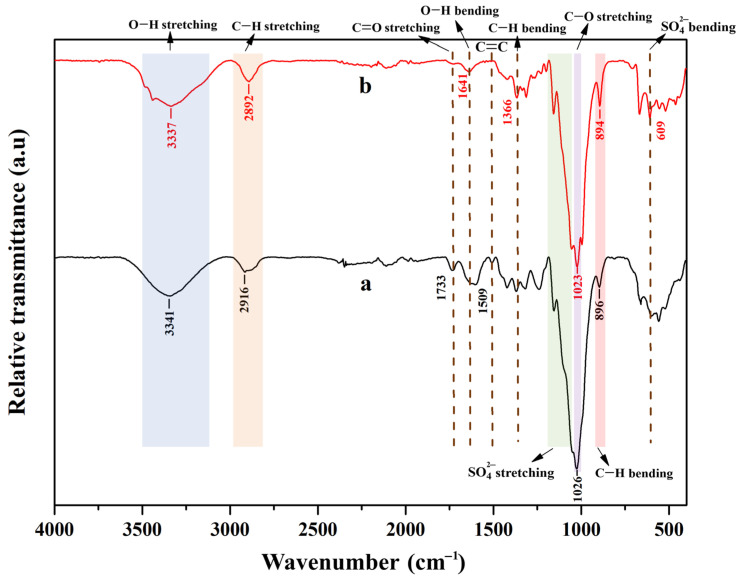
FT-IR spectra of cellulose from hemp: (a) nano-grinded hemp and (b) cellulose nanocrystals.

Cellulose fibers were successfully extracted and purified from hemp. A yellowish fine powder with a uniform size was obtained. It can be dispersed in DI water as a suspension. Figure 1 shows the FT-IR spectra of cellulose. The spectrum of pristine hemp is also provided for comparison. No significant change in the peak position or its intensity was observed. This shows that the alkaline reaction and sulfuric acid treatment did not have any effect on the functional group of cellulose nanocrystals. The broad peak in the region 3300 to 3500 cm^−1^ is attributed to the O–H stretching vibration. The peaks at 2916 cm^−1^ and 2892 cm^−1^ for hemp and cellulose nanocrystals, respectively, correspond to the C–H stretching vibrations. The spectra of the cellulose nanocrystals and hemp showed the characteristics of O–H stretching and C–H stretching vibrations [16]. The peak at 1733 cm^−1^ is consistent with the C=O stretching vibrations and can be attributed to the carboxyl and acetyl groups of hemicellulose. This peak disappeared in the CNC spectra. Therefore, this confirms the absence of hemicellulose in cellulose nanocrystals [17] after alkaline treatment. A peak at 1641 cm^−1^ was observed in the obtained cellulose fibers after treatment and extraction into CNCs. It is attributed to the O–H bending of the adsorbed water [16]. The peak at 1509 cm^−1^ corresponds to the C=C vibrations of the hemp before treatment. Furthermore, the band at 1366 cm^−1^ in CNCs was associated with the C–H bending vibration and C–O bonds in the polysaccharide rings [18], and the peaks at 1023 to 1026 cm^−1^ also relate to the C–O stretching vibration of the polysaccharide [19]. The peaks between 1192 cm^−1^ and 1072 cm^−1^ correspond to the symmetrical vibration of SO_4_^2−^. In addition, the peak at 609 cm^−1^ is due to the SO_4_^2−^ out-of-plane bending vibration [20]. An increased intensity of the peak at 894 cm^−1^ of CNCs was observed as compared to the peak at 896 cm^−1^ of hemp, and this can be attributed to the C–H bending vibration of cellulose I, which is the native cellulose [21].

**Figure 2:**
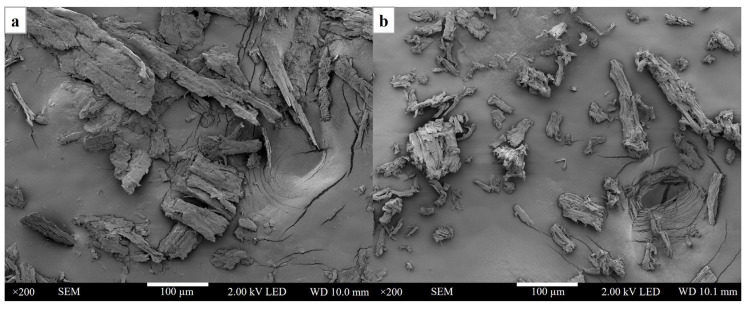
The microstructure of cellulose from hemp: (**a**) nano-grinded hemp and (**b**) cellulose nanocrystals.

Figure 2 reports the microstructure of cellulose prepared from hemp. Figure 2a,b exhibit the morphological properties of hemp after the nano-grinding process and the transformation of its cellulose fibers to cellulose nanocrystals. The morphology of pristine hemp can be characterized as large particles with a non-uniform dispersion. After sulfuric acid hydrolysis, the size of the cellulose nanoparticles was typically smaller and uniformly distributed. The smaller size of cellulose nanoparticles is essential for reinforcement in composite materials. In addition, the hemicellulose and lignin were eliminated after the chemical and modification processes [22].

### 3.2. Fabrication of Cellulose Derived from Hemp and Polyvinyl Alcohol-Based Hydrogel Composite

Cellulose nanocrystal and polyvinyl alcohol-based composite hydrogel was successfully prepared. The composite hydrogel exhibited the characteristics of stretchability and reformability under an external applied load. The hydrogel can retain a high number of water molecules within the hydrogel network. Hydrogel with a high number of cellulose nanocrystals exhibited low stretchability. This could be due to extensive hydrogel bonding interactions throughout the hydrogel network. As a result, an increase in the mechanical properties of the hydrogel was observed. Furthermore, as expected, the composite hydrogel exhibited high hydrophilicity and absorbed water quickly. Borax can be employed as a crosslinking agent for its ability to form hydrogen bonding interactions as well as dynamic covalent bond formation throughout the hydrogel network [23,24,25,26]. Therefore, the hydrogels have a strong self-healing property as well as excellent mechanical strength [27].

**Figure 3:**
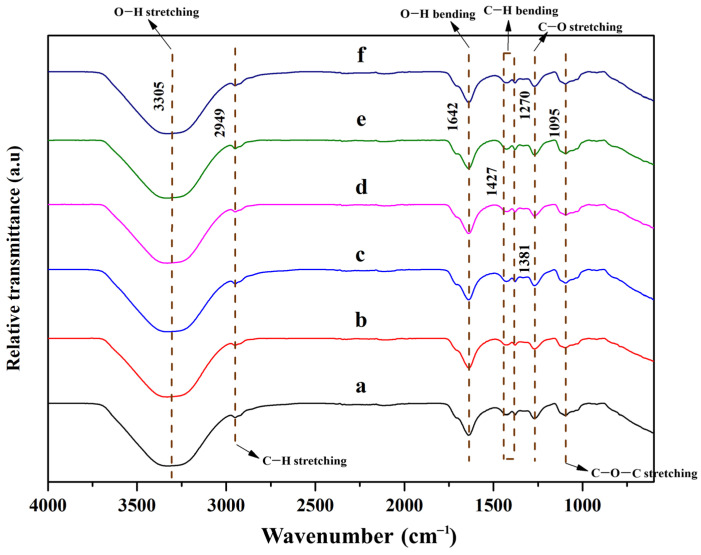
FT-IR spectra of cellulose nanocrystals from hemp and polyvinyl alcohol-based composite hydrogel: (a) pristine PVA, (b) 1% CNC/PVA, (c) 3% CNC/PVA, (d) 5% CNC/PVA, (e) 7% CNC/PVA, and (f) 10% CNC/PVA.

Figure 3 reports the FT-IR spectra of cellulose nanocrystals and polyvinyl alcohol-based composite hydrogels. Only 1–10% (*w*/*v*) of CNCs in composite hydrogels were investigated. The peak of the pristine matrix is also provided for comparison. No significant change in peaks was observed, as compared to the pristine PVA matrix.

The characteristic peaks at 3305 cm^−1^ and 1642 cm^−1^ are attributed to O–H stretching and O–H bending, respectively [28]. These are typically related to hydroxyl groups and are responsible for the hydrogen bonding throughout the hydrogel network. The peaks at 2949 cm^−1^, 1427 cm^−1^, and 1381 cm^−1^ are assigned to the C–H bond. These were assigned to symmetric, asymmetric, and bending vibrations characteristic of C–H bonding. The peak at 2949 cm^−1^ is assigned to C–H stretching and attributed to the alkyl chains of polyvinyl alcohol [29]. On the other hand, the peaks at 1270 cm^−1^ and 1095 cm^−1^ are attributed to C–O stretching and C–O–C stretching, respectively. These groups are referred to as glycosidic bonds between D-glucose units within the cellulose structure [30]. This confirms that cellulose nanocrystals are successfully embedded into the matrix. In the case of the 1–10 wt% of CNC/PVA hydrogel, the O–H stretching vibration peak shifted in the region of 3300–3500 cm^−1^ due to the intermolecular hydrogen bonds [31].

**Figure 4:**
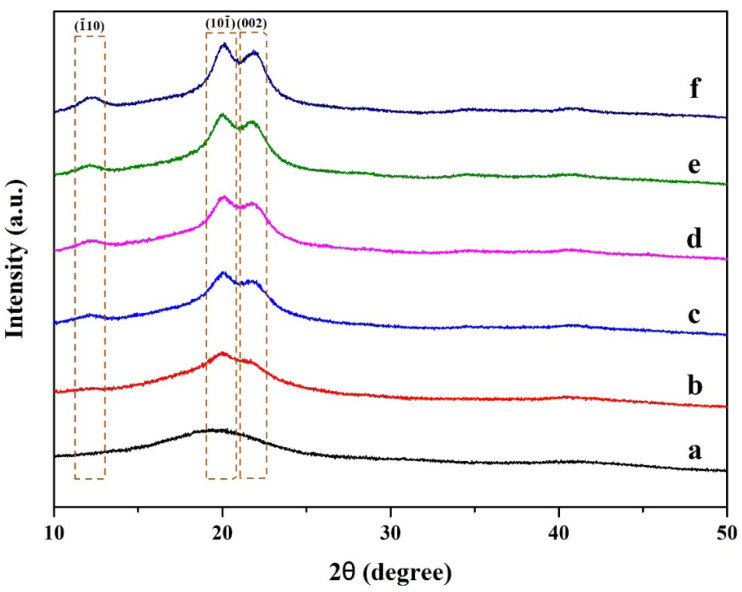
XRD pattern of cellulose nanocrystals from hemp and polyvinyl alcohol-based composite hydrogel: (a) pristine PVA, (b) 1% CNC/PVA, (c) 3% CNC/PVA, (d) 5% CNC/PVA, (e) 7% CNC/PVA, and (f) 10% CNC/PVA.

Figure 4 shows the XRD profile of cellulose nanocrystal and polyvinyl alcohol-based composite hydrogel. The XRD pattern of pristine PVA is shown in Figure 4a, and the composite hydrogels are shown in Figure 4b–f. All of the characteristic peaks have similar features, indicating polyvinyl alcohol as the hydrogel matrix. It should be noted that the CNCs are embedded inside the PVA matrix. No cellulose nanocrystals were observed on the PVA surface. With the increase in cellulose nanocrystal content, a stronger peak was observed. It can be explained that pristine PVA exhibited an amorphous phase, while cellulose nanocrystals exhibited a crystalline phase. It was observed that when the cellulose nanocrystals were integrated, the presence of crystallinity was enhanced. From Figure 4, these characteristic peaks were observed. These peaks were presented at the diffraction angles of 12.2°, 20.1°, and 22°. These angles were indexed to diffraction planes of ($\bar{1}$10), (10$\bar{1}$), and (002), respectively. These peaks are attributed to purified cellulose, as suggested by previous reports [32]. This confirms that alkali treatment and acid hydrolysis can be used to access the single phase of cellulose, and other impurities are successfully removed.

**Figure 5:**
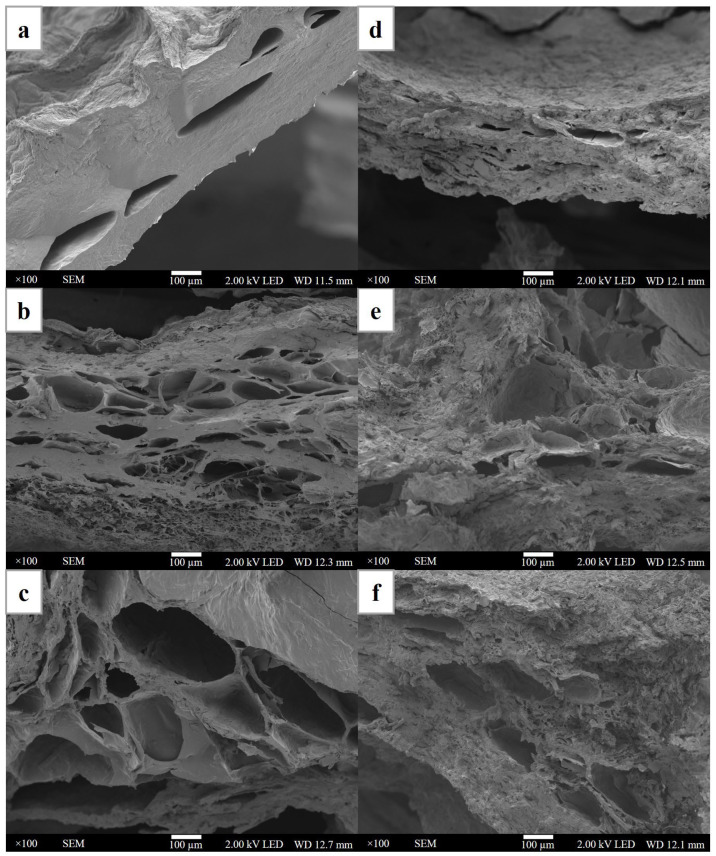
The microstructure of cellulose nanocrystals from hemp and polyvinyl alcohol-based composite hydrogel: (**a**) pristine PVA, (**b**) 1% CNC/PVA, (**c**) 3% CNC/PVA, (**d**) 5% CNC/PVA, (**e**) 7% CNC/PVA, and (**f**) 10% CNC/PVA. A cross-sectional view with a magnification of 100× was reported.

Figure 5 shows the morphology of cellulose nanocrystal and polyvinyl alcohol-based composite hydrogels. The pristine PVA showed a dense and smooth structure with a large pore size. The composite hydrogels showed a smaller pore size and displayed a rough structure with an increased number of micropores. With increasing CNC content, the pore sizes were found to be reduced. Strong hydrogen bonding interactions throughout the network could explain the smaller pore sizes with increasing CNC content, as suggested by de Lima et al. [33]. With the smaller pore size and the formation of micropores, the superiority of the compressive strength was observed under an applied external load [34]. Furthermore, the composite hydrogel with a large number of micropores in the structure could be suitable for cell growth and proliferation. The micropores can allow the transport of gases, nutrients, and soluble factors, as well as waste removal from the cell [35]. Furthermore, it was observed that the cellulose nanocrystals were inserted in between the polyvinyl alcohol matrix. With a high cellulose nanocrystal content, large interface structures were noted, which also accounts for the porosity and cracks. The porous structure of CNC/PVA composites allowed a large amount of water to penetrate into the matrix, as noted by the high swelling behavior and degradation [36,37].

**Figure 6:**
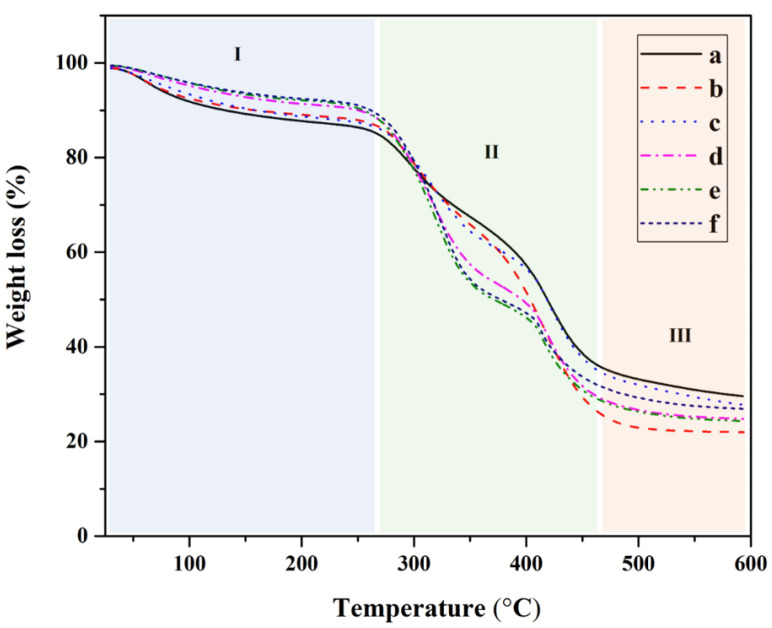
TGA thermogram of cellulose nanocrystals from hemp and polyvinyl alcohol-based composite hydrogel: (a) pristine PVA, (b) 1% CNC/PVA, (c) 3% CNC/PVA, (d) 5% CNC/PVA, (e) 7% CNC/PVA, and (f) 10% CNC/PVA.

The thermal stability of composite hydrogel is considered as one of the important characteristics of the hydrogel. Figure 6 shows the thermal decomposition of cellulose nanocrystal and polyvinyl alcohol-based composite hydrogels. Pristine PVA was also analyzed for comparison purposes. All of the curves show a similar profile. The TGA thermogram can be classified into three distinct regions based on temperature elevation. The first part of the weight loss (%) was considered from room temperature to 260 °C, and a weight loss of ~13.5% was observed. This can be attributed to the moisture content in the composite hydrogel [38]. Then, 38.4% of the weight loss in the second region was observed between 260 and 460 °C. This wide range can be attributed to the decomposition of cellulose nanocrystals and polyvinyl alcohol [39]. It can be observed that, in the presence of cellulose nanocrystals, thermal resistance was slightly increased. This is probably due to the fact that the cellulose nanocrystals exhibited high thermal resistivity [40]. When the temperature was increased to above 460 °C, the samples were completely degraded. The remaining amounts for all compositions, 26% or less, were attributed to residues and char.

**Figure 7:**
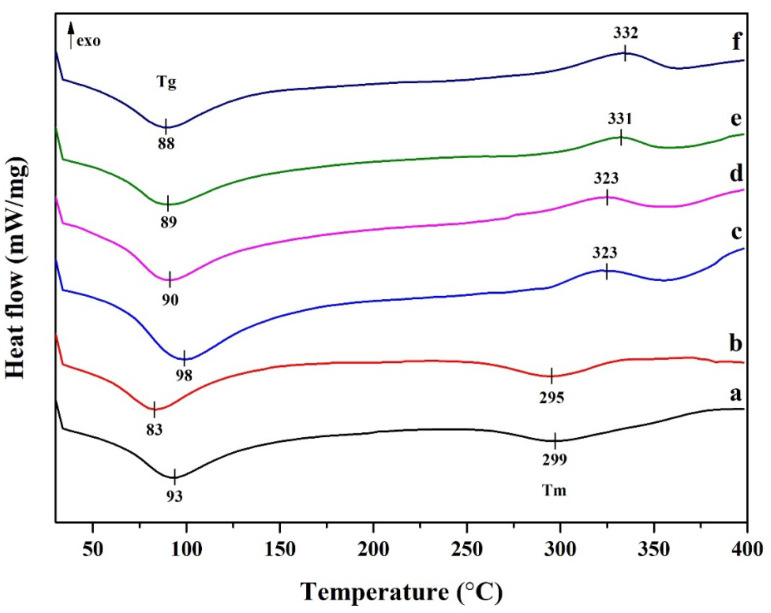
DSC thermogram of cellulose nanocrystals from hemp and polyvinyl alcohol-based composite hydrogel: (a) pristine PVA, (b) 1% CNC/PVA, (c) 3% CNC/PVA, (d) 5% CNC/PVA, (e) 7% CNC/PVA, and (f) 10% CNC/PVA.

Figure 7 shows the DSC curves, where the pristine PVA is shown in Figure 7a, while the DSC curves of the CNC/PVA composites are shown in Figure 7b–f. The glass transition temperatures (T_g_) of all compositions were between 83 and 98 °C, with endothermic peaks. The glass transition temperatures of the composite hydrogels did not change significantly with different CNC content. This could be due to the strong hydrogen bonding interactions between the polymer chains and the low content of CNCs in the hydrogel. In addition, the melting temperatures of the composite hydrogels exhibit exothermic peaks between 323 °C and 332 °C. The melting temperatures were found to increase with increasing cellulose nanocrystal content, similar to a previous report [41]. Similar curves were obtained for the hydrogel made with 1% CNC filled into PVA matrix and the pristine PVA.

### 3.3. Preliminary Investigation as a Biomaterial

The composite hydrogels can be applied as a wound dressing. An effective wound dressing is characterized by its ability to adsorb excessive exudate from wounds and also prevent complete dehydration of the wound for efficient cell attachment and proliferation [42].

**Figure 8:**
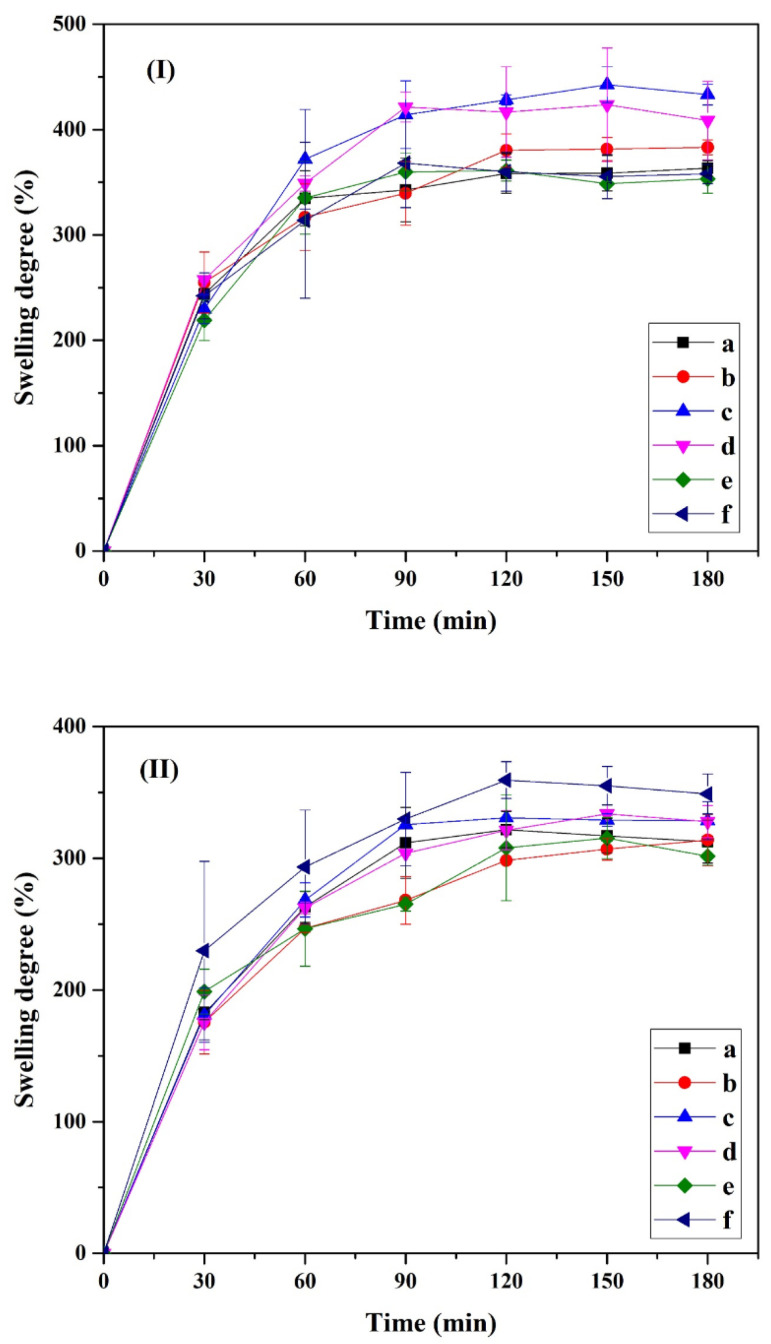
Swelling behavior of cellulose nanocrystals from hemp and polyvinyl alcohol-based composite hydrogel: (a) pristine PVA, (b) 1% CNC/PVA, (c) 3% CNC/PVA, (d) 5% CNC/PVA, (e) 7% CNC/PVA, and (f) 10% CNC/PVA with (**I**) DI water and (**II**) PBS (pH 7.4) at 37 °C.

Figure 8 shows the swelling behavior of CNC-based composite hydrogel. The swelling behavior of the composite hydrogel was investigated in both deionized water and PBS solution at 37 °C. The swelling behaviors at the initial stage (between 0 and 30 min) were very rapid due to the highly hydrophilic nature of the hydrogels [43,44]. Then, the swelling rate was constant after 180 min. The swelling behavior of the composite hydrogel corresponded to the number of cellulose nanocrystals. The swelling was found to increase with increasing CNC content in the PVA matrix; this could be due to more hydrogen bonding interactions with water molecules with increasing CNC content [33,45]. In addition, the pore size within the network structure of the composite hydrogel was found to increase, similar to in a previous report by Kaushik et al. [45]. During the investigation of swelling behavior, it was remarkable to note that the composite hydrogels in PBS solution were more stable than those in DI water. This could be due to stronger electrostatic interactions due to the presence of anionic CNC [46].

The gel fraction percentages of the composite hydrogels are shown in Table 1. It is noted that the gel fraction values are dependent on the CNC content. With increasing CNC content, more hydrogen bonding interactions are expected within the cellulose nanocrystal and PVA network. Furthermore, the gel fraction is also dependent on the density of crosslinking in the gel. It is observed that the chain entanglement and restrictions between cellulose nanocrystals and PVA chains can be enhanced [47]. It should be noted that the gel fraction values of different composite hydrogels in PBS solution were higher than those in DI water. Stronger electrostatic interactions in the gel could explain the higher stability [48].

**Figure 9:**
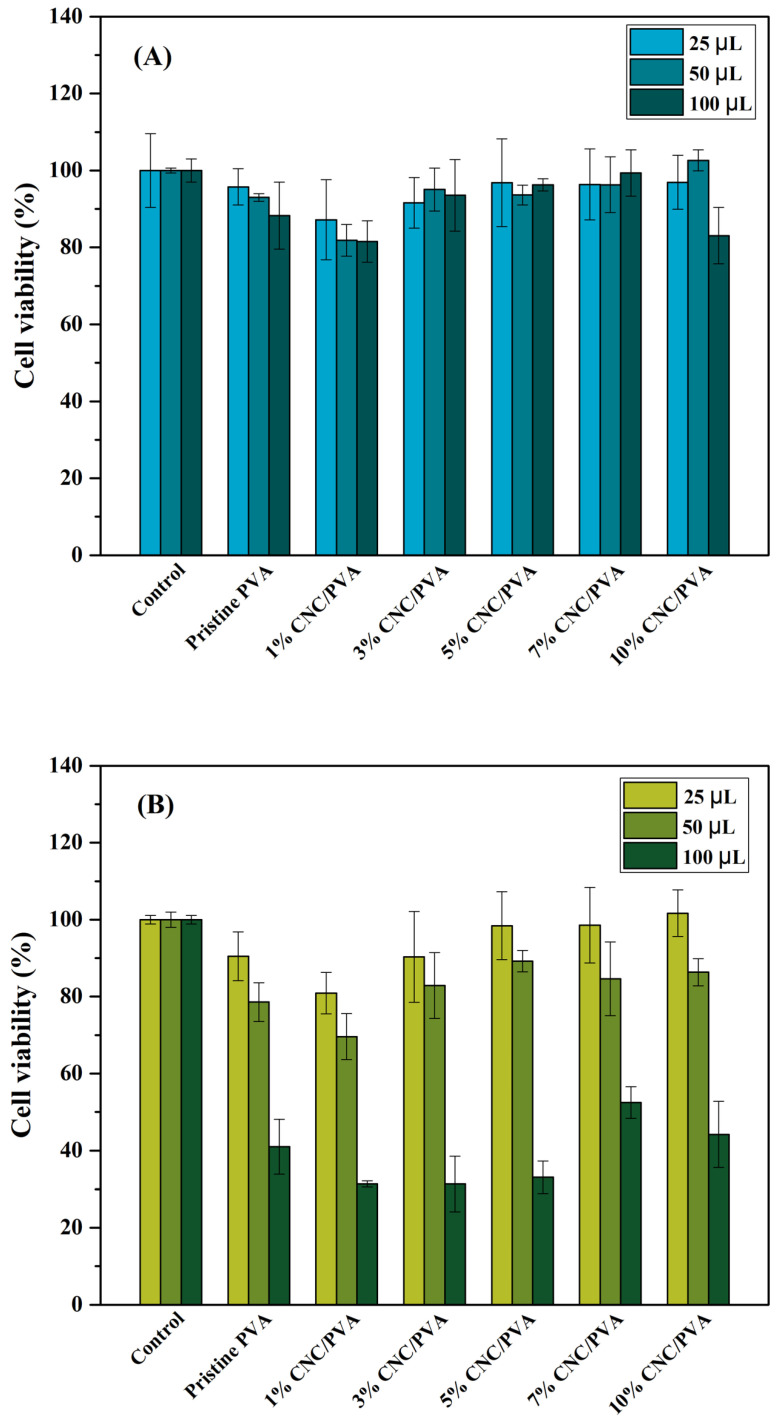
Cytotoxicity of cellulose nanocrystals from hemp and polyvinyl alcohol-based composite hydrogel extract at 25, 50, and 100 µL cultured with (**A**) cancer cells (Hela) and (**B**) fibroblast cells (HDFa).

Cytotoxicity evaluation is essential for determining the biocompatibility of the prepared composite hydrogels. Figure 9 shows the cell viability of cancer cells (Hela) and fibroblast cells (HDFa) after they were cultured with various CNC/PVA composite hydrogel extracts at 25, 50, and 100 µL for 24 h. The MTT assay was used to evaluate cell viability of the samples. The results in Figure 9A show that the addition of increasing concentrations of cellulose nanocrystals into the PVA hydrogel and the extraction of composite hydrogel at different concentrations did not negatively affect Hela cells compared to the pristine PVA hydrogel. According to the cell viability results for Hela cells, all hydrogel extracts reach more than 70%. It was confirmed that the different numbers of hemp-derived cellulose nanocrystals integrated into PVA hydrogel were non-toxic hydrogels [49]. Furthermore, the viability of HDFa cells cultured with the same amount of hydrogel extracts (25, 50, 100 µL) was revealed to be 69.6–101.6% at 25 and 50 µL, respectively. This result shows that the prepared composite hydrogels have low toxicity to HDFa cells. However, with 100 µL of hydrogel extract, some cytotoxic effects were noted with fibroblast HDFa cells. This can be due to the high amount of PVA after degradation in the 100 μL extract, which can impact osmotic pressure, resulting in cell rupture and death [50]. The CNC/PVA composite hydrogels have been shown to be a potential biomaterial, such as for wound healing, due to their non-toxic nature and biocompatibility [51].

## 4. Conclusions

Cellulose nanocrystals from hemp and polyvinyl alcohol-based composite hydrogels were prepared through chemical crosslinking by sodium tetraborate, also known as borax. The hydrogel scaffold was freeze-dried to form a porous structural morphology. XRD confirmed the presence of cellulose nanocrystals and polyvinyl alcohol in the composite hydrogel. With increasing CNC content, the size of the pores tends to decrease. Furthermore, it was observed that the swelling characteristic is very high at the initial stage, and then it reached a plateau region. Furthermore, the cellulose nanocrystals slightly enhanced the thermal stability of the composite hydrogel. The glass transition temperature of the composite hydrogel did not change. The cell viability in different amounts of composite hydrogel extract at 25, 50, and 100 µL with Hela cells was found to be higher than 70%, which shows the low toxicity of the composite hydrogel. For HDFa cells, similar toxicity results were obtained, with the exception of 100 µL. With favorable properties such as thermal stability, swelling behavior, and biocompatibility, the 3% CNC/PVA composite hydrogel will be considered for further development in our future work.

## Figures and Tables

**Table 1:** Percentage of gel fraction of the prepared cellulose nanocrystals from hemp and polyvinyl alcohol-based composite hydrogel.

Sample	Gel Fraction (%)	
	DI	PBS
Pristine PVA	68.389 ± 1.176	85.969 ± 2.452
1% CNC/PVA	66.022 ± 6.022	87.235 ± 3.192
3% CNC/PVA	64.980 ± 3.442	90.734 ± 6.040
5% CNC/PVA	59.076 ± 3.424	88.531 ± 2.123
7% CNC/PVA	64.906 ± 2.115	85.892 ± 2.021
10% CNC/PVA	62.653 ± 1.115	85.637 ± 1.983

## Data Availability

Data is available from the authors upon request.
